# Drug-related problems and its determinant among hospitalized neonates with sepsis at Jimma University Medical Center, Ethiopia: a prospective observational study

**DOI:** 10.1186/s40780-021-00203-0

**Published:** 2021-06-01

**Authors:** Mengist Awoke, Tsegaye Melaku, Mohammed Beshir

**Affiliations:** 1 Department of Clinical Pharmacy, School of Pharmacy, Institute of Health, Jimma University, PO. Box 378, Jimma, Ethiopia; 2 Department of Pediatrics, Jimma University Medical Center, PO. Box 378, Jimma, Ethiopia

**Keywords:** Neonate, Drug-related problem, Sepsis, Neonatal intensive care unit, Ethiopia

## Abstract

**Background:**

Neonatal populations are quite susceptible to drug-related problems (DRPs) because of clinical heterogeneity and clinical practice trends. However, studies reporting DRPs in the neonatal population are quite limited.

**Objective:**

This study aimed to assess the magnitude and types of DRPs and determinant factors among neonates admitted with neonatal sepsis at the Neonatal Intensive Care Unit (NICU) of the Jimma University Medical Center (JUMC), Ethiopia.

**Methods:**

A hospital-based prospective observational study was conducted involving 201 neonates with sepsis admitted to the NICU from May to August 30, 2018. DRPs were classified using Cipolle’s classification method. Statistical Package for Social Science Version 22 was employed for data analysis. Logistical regression was carried out to determine the determinants of DRPs. A *p*-value < 0.05 was considered to be statistically significant.

**Results:**

Of 201 neonates with sepsis included in this study, 125 (62.2%) were males and the median age of the neonate was 5 days. The mean (±standard deviation) number of medications taking during their hospital stay was 2.6 ± 0.7. DRPs were identified in 98 neonates, at a rate of 48.8% (95% CI, 41.7–55.9). Dose too high (42, 35.8%) and need additional drug therapy (40, 34.1%) were the commonly identified DRPs. Taking antibiotics plus other medications (Adjusted Odds Ratio (AOR) =5.2, 95%CI [1.2–22.0], *p* = 0.02) was a determinant factor for the occurrence of DRPs.

**Conclusion:**

The burdens of DRPs occurrence were high in hospitalized neonates with sepsis. The most common DRPs identified were dose too high and need additional drug therapy. Combined use of other medicines with antibiotics was a predictor of DRP occurrence. The innovative way to tackle the occurrence of DRPs, such as the incorporation of clinical pharmacy service provider into the neonatal care team, which will prevent, detect and/or minimize the occurrence of DRPs, is highly recommended.

**Supplementary Information:**

The online version contains supplementary material available at 10.1186/s40780-021-00203-0.

## Background

The neonatal period is defined as age from birth to one month of life [1]. Because of the immaturity of their immune system, neonates tend to become infected easily. Thus, there is a likelihood of developing a serious infection such as neonatal sepsis, which requires pharmacological and non-pharmacological intervention [2]. Many patients do not receive the intended beneficial effects of their treatment as a result of DRPs [3]. It is defined as a drug-related event or circumstance that actually or potentially interferes with desired health outcomes and it is classified into seven domains to support the assessment of indication, effectiveness, safety, and adherence [4, 5]. The domains include unnecessary drug therapy, the need for additional drug therapy, ineffective drug, dosage too low, dosage too high, adverse drug reactions, and non-compliance [5].

When DRPs are not recognized and/or resolved, patients may experience treatment failure, adverse events, sub-optimal quality of life, and facing new medical conditions as well as mortality [3, 6, 7]. Furthermore, problems involving medication are associated with emergency department visits, extended hospitalizations, additional visits to the physician’s office, and additional prescriptions [8, 9]. Overall, medication-related morbidity accounts for 7.0% of hospitalizations and has led to unnecessary health care expenditures in billions of dollars [10].

Neonates, in particular, are quite susceptible to DRPs because of clinical heterogeneity (i.e. rapid body growth/weight change, gestational age, postnatal age, and physiological immaturity) as well as clinical practice patterns such as dose calculation as a function of body weight [11–13].

The occurrence of DRPs in the pediatric population varies from 25.6 to 59.8% [14–18]. Though the occurrence rate of DRPs is more frequent in the intensive care unit (ICU) due to the complicated medical condition and the associated complexity of drug therapy [19], studies assessing it on hospitalized neonatal are highly limited [14, 17]. In Ethiopia, a study found that the extent of DRPs in infectious disease patients was as high as 70.7% [20] and studies have shown that neonatal sepsis is the most common reason for admission to the NICU in the range of 22.7–28.6% [21, 22] and cause of death between 4.1 and 18.4% [22, 23]. Despite this, no previous studies have addressed the magnitude of DRPs and their impact on neonates hospitalized at the NICU in Ethiopia. Thus, this study proposed to explore the magnitude and types of DRPs, and its determinant factors among neonates admitted with sepsis at the NICU of JUMC, Jimma, Southwest of Ethiopia.

## Methods

### Study design and setting

A hospital-based prospective observational study was conducted from May to August 30, 2018, at the NICU of JUMC, a tertiary teaching hospital in Jimma town, Jimma zone, Oromia region, Southwest of Ethiopia. It is located 352 km from Addis Ababa, the capital. The neonatal care unit provides service for both inborn and outborn babies. The unit has resuscitation facilities, incubators, radiator heater, and phototherapy unit.

### Eligibility criteria

#### Inclusion criteria

- ✓ Neonates age < one month and admitted with the physician diagnosis of neonatal sepsis (either early-onset (EONS) or late-onset neonatal sepsis (LONS)) at NICU.
- ✓ The neonate’s guard/parent giving written consent.

#### Exclusion criteria

- ✓ Neonates diagnosed with meningitis based on the clinical finding and/or Cerebrospinal fluid (CSF) analysis (i.e. Cerebrospinal fluid white blood cell (WBC) counts > 20 cells/mm^3^).

### Sample size

The sample size was computed with the following assumptions made, a 5% margin of error, 95% confidence interval, a 50% prevalence of drug-associated problems among neonates, an estimated source population size of 380, and adding a non-response rate of 5%. Finally, the computed sample size was 201 neonates admitted with sepsis at the NICU.

### Data collection instrument and process

The data abstraction tool was developed following a review of the neonate’s medical charts and various literatures. Two data collectors (pharmacists) were trained and collected the data daily over 4 months (from May to August 30, 2018) following the pretest. The patients’ medical charts and physician medication order sheets were used as a data source. Data on sex, gestational age, birth weight, neonatal age, types of sepsis, clinical characteristics, lab findings, comorbidity, medications taken, and discharge status were retrieved from the neonate’s medical chart. While data extracted from the physician’s medication order sheet includes the drug’s name, dose, frequency, duration, and the number of prescribed drugs. For each neonate, the medical conditions and prescribed drugs were cross-checked and assessed to identify the presence of potential DRPs based on details from neonatal standard guidelines including Ethiopian Standard Treatment Guidelines [24], World Health Organization (WHO) neonatal treatment guideline 2016 and 2017 [25–27], American Academy of Pediatrics [28], and Neonatology A Practical Approach to Neonatal Diseases [29]. The identified DRPs were recorded on the pharmacotherapy follow-up sheets daily throughout the hospital stay, which was reviewed again independently by a Clinical Pharmacist and a Pediatrician and forwarded the final judgment based on their agreement and per the available guideline. The identified DRPs were classified by using a Cipolle’s method [5]; as unnecessary drug therapy, needs additional drug therapy, ineffective drug therapy, dosage too low, dosage too high, adverse drug reaction, and noncompliance.

### Data quality assurance

The data abstraction tool has been translated from English into the dominant local languages (Amharic and Afaan Oromo) and translated back into English by an independent person to ensure consistency. The tool was pre-tested before commencing the actual data collection and then the necessary adjustment was performed. Before data entry and analysis, data were cleared, categorized, compiled, coded, and verified to ensure completeness and accuracy.

### Data processing and analysis

Data were entered into Epi data 4.2 and then exported to Statistical Package for Social Science (SPSS) 22 for analysis. For continuous data, the normal test was performed using the Shapiro-Wilk’s test. For this purpose, a level of significance of 0.05 was used. Parametric data were reported with a mean (±standard deviation), whereas non-parametric data were reported with the median. Frequency and percentage were computed for categorical variables and a chi-square test was conducted to check for cell adequacy. Bivariate logistic regression was carried out to investigate associations between the DRPs and independent variables. Then, a backward, stepwise multivariate logistic regression [reported with Adjusted odds Ratios (AOR) with 95% Confidence Intervals (95% CI] was performed including all explanatory variables with a *p*-value of < 0.25 on bivariate logistic regression to evaluate determinants of DRPs. A *p*-value < 0.05 was considered to be statistically significant.

### Definition of terms

#### Neonatal sepsis

Clinically suspected systemic infection characterized by the presence of danger signs such as poor feeding, convulsions, drowsiness or unconsciousness, the movement only when stimulated or no movement at all, fast breathing ≥60 breaths/min, grunting, severe chest in-drawing, raised temperature > 37.5 °C, hypothermia < 35.5 °C or central cyanosis during the first one month of life [30].

#### Drug-related problem

Drug-related event or circumstance that actually or potentially interferes with desired health outcomes [4, 5].

Category of DRPs was defined and identified based on Cipolle’s method [5] as follows:

- ➢ Need for additional drug therapy was considered when:
-  ✓ The medical condition requires the initiation of drug therapy.
-  ✓ Preventive drug therapy is required to reduce the risk of developing a new condition.
-  ✓ The medical condition requires additional drug therapy to achieve synergistic or additive effects.
- ➢ Ineffective drug therapy was considered when:
-  ✓ The drug is not the most effective for the medical condition and a different drug is needed.
-  ✓ The medical condition is refractory to the drug product and a different drug is needed.
-  ✓ The drug product is contraindicated in this patient.
-  ✓ The dosage form of the drug product is inappropriate
-  ✓ The drug product used is not an effective product for the medical condition being treated.
- ➢ Unnecessary drug therapy is considered when:
-  ✓ There is no valid medical indication for the drug therapy at this time,
-  ✓ Multiple drug products are being used for a condition that requires single-drug therapy.
-  ✓ The medical condition is more appropriately treated with nondrug therapy,
-  ✓ The drug therapy is used to treat an avoidable adverse reaction associated with another medication.
- ➢ Dosage too high was considered when:
-  ✓ The dose of the drug is too high for the patient, resulting in toxicity.
-  ✓ The dosing frequency is too short,
-  ✓ The duration of drug therapy is too long,
-  ✓ The drug interaction occurs that could result in a toxic reaction to the drug product.
-  ✓ The dose of the drug was administered too rapidly.
- ➢ Dosage too low was considered when:
-  ✓ The dose of the drug is too low to produce the desired response.
-  ✓ The drug interaction occurs that could reduce the amount of active drug available,
-  ✓ The duration of drug therapy is too short to produce the desired response,
-  ✓ The dosing interval is too short to produce the desired response.
- ➢ Adverse drug reaction was considered when:
-  ✓ The drug causes an undesirable reaction that was not dose-related,
-  ✓ The drug interaction causes an undesirable reaction that is not dose-related,
-  ✓ Incorrect administration: the drug product was administered by the incorrect route or method resulting in an adverse reaction.
-  ✓ The drug causes an allergic reaction.
-  ✓ Dosage increase/decrease too fast: the drug dosage was administered or escalated too rapidly resulting in an adverse reaction.
-  ✓ Unsafe drug for the patient: a safer drug product is required due to patient risk factors.
- ➢ Noncompliance/non-adherence is considered if a patient fails to take medications appropriately due to one of the following reasons:
-  ✓ Lack of understanding the instructions,
-  ✓ Preference not to take the medication,
-  ✓ Forgetfulness,
-  ✓ Inability to swallow or self-administer the drug product appropriately,
-  ✓ Affordability and availability problem.

## Results

### Study participant’s enrollment information

Two-hundred ten neonates with sepsis were assessed for eligibility. Eight neonates were excluded due to CSF analysis suggesting meningitis and one of the newborn’s parents/caregivers declined to participate in the study. Finally, two-hundred one neonates with sepsis were included in the analysis.

### Socio-demographic and clinical characteristics

The majority of neonates in this study were males 125 (62.2%). The mean (±SD) birth weight and gestational age was 2.7 ± 0.7 kg, and 37 ± 2.1 weeks, respectively. The median age of the neonate was 5 days. Among clinical features, most neonates had poor feeding 182 (90.5%) followed by grunting (55.2%) and respiratory distress (35.8%) on admission. The mean (±SD) body temperature, respiratory rate, and heart rate on admission was 37.3 ± 1.3, 61.9 ± 14.4 breaths/minute, and 143.8 ± 15.6 beats/minute, respectively. One hundred twenty-two (60.6%) neonates were diagnosed with early-onset neonatal sepsis (EONS) (Table 1).

**Table 1:** Socio-demographic and clinical characteristics of neonates admitted with neonatal sepsis

Variables	Frequency (%)
Gender	
Male	125 (62.2)
Female	76 (37.8)
Birth weight (mean ± SD) (kg)	2.7 ± 0.7
On diagnosis weight (mean ± SD) (kg)	3.1 ± 1.0
Age (median)	5 days
Gestational age (mean ± SD) (weeks)	37 ± 2.1
Poor feeding	182 (90.5)
Diarrhea	13 (6.5)
Convulsion	41 (20.4)
Vomiting	46 (22.9)
Respiratory distress	72 (35.8)
Grunting	111 (55.2)
Cyanosis	9 (4.5)
Jaundice	19 (9.5)
Body temperature (mean ± SD) (°c)	37.3 ± 1.3
Respiratory rate (mean ± SD) (breath/minute)	61.9 ± 14.4
Heart rate (mean ± SD) (beat/minute)	143.8 ± 15.6
Neonates with comorbidity	96 (47.8)
Types of sepsis	
EONS	122 (60.6)
LONS	79 (39.4)

*°c* Celsius/Centigrade, *EONS* Early Onset Neonatal Sepsis, *LONS* Late Onset Neonatal Sepsis, *SD* Standard Deviation

### Baseline laboratory findings

At the time of admission, the mean (±SD) number of white blood cells and red blood cells was 13,939.0 ± 73.0cells/mm^3^ and 4.1 × 10^6^ ± 1.1 × 10^6^ cells/mm^3^, respectively. The average blood sugar and oxygen saturation levels of the study population were 113.9 ± 64.3 g/dL and 80.4 ± 11.3%, respectively (Table 2).

**Table 2:** Baseline laboratory findings of neonates with sepsis

Laboratory parameters	Value (Mean ± SD)
White blood cells (cell/mm^3^)	13,939.0 ± 73.0
Red blood cells (cell/mm^3^)	4.1 × 10^6^ ± 1.1 × 10^6^
Hemoglobin (g/dL)	14.1 ± 3.8
Platelet count (cell/mm^3^)	252,786 ± 174,104
Blood glucose (mg/dL)	113.9 ± 64.3
Oxygen saturation (%)	80.4 ± 11.3

*g* gram, *dL* deciliter, *SD* Standard Deviation

### Medication-related factors

The mean (±SD) number of medications taken during the hospital stay was 2.6 ± 0.7. Two hundred ninety-four prescriptions were ordered with a total of 530 medications (i.e. 1.81 medications per each prescription). Ampicillin plus gentamicin (188, 93.5%) had been the most frequently used antibiotics for the management of neonatal sepsis. Ninety-three neonates were taking non-antibiotic medications, of which, paracetamol (17, 18.3%) followed by phenobarbital (11, 11.8%) and maintenance fluid (10, 10.8%) were used by most of them (Table 3).

**Table 3:** Medications used for the management of the neonatal sepsis

Variables		Frequency (%)
Number of medications taking during their hospital stay		2.6 ± 0.7^a^
Antibiotics used (*n* = 201)	Ampicillin + Gentamicin	188 (93.5)
	Ceftriaxone + Gentamicin	9 (4.5)
	Ampicillin + Ceftriaxone	3 (1.5)
	Ceftazidime + vancomycin	1 (0.5)
Non-antibiotic medications used (*n* = 93)	Paracetamol	17 (18.3)
	Dextrose	14 (15.0)
	Phenobarbital	11 (11.8)
	Maintenance Fluid + Blood transfusion	10 (10.8)
	Vitamin K	5 (5.4)
	Iron + Folic Acid	5 (5.4)
	Cimetidine	5 (5.4)
	Paracetamol + Vitamin K	5 (5.4)
	Paracetamol + Phenobarbital	4 (4.4)
	Phenytoin + Phenobarbital	4 (4.4)
	Phenobarbital +Calcium Gluconate	4 (4.4)
	Phenobarbital + Vitamin K	3 (3.2)
	Insulin	3 (3.2)
	Others	3 (3.2)

^a^mean ± standard deviation

### Drug related problems identified among neonates with sepsis

Among the study population, a total of 121 DRPs were identified in 98 neonates (at least 1 DRP per neonates) with a rate of 48.8% [95% CI, 41.7–55.9]. Dose too high (42, 34.7%) followed by need an additional drug therapy (40, 33.5%) and dose too low (24, 19.8%) were the commonly identified DRPs. Antibiotics (52, 42.9%) were the most commonly implicated drug category in DRPs, followed by analgesics-antipyretics (29.7%) and antiepileptic drugs (14.0%) (Table 4).

**Table 4:** Drug-related problems (DRPs) identified and classes of medications involved in the occurrence of DRPs among neonates with sepsis

Variables			Frequency (%)
Neonates with drug-related problem			98 (48.8)
Drug-related needs and classification of DRPs	Indication	Unnecessary drug therapy	7 (5.7)
		Need an additional drug therapy	40 (33.5)
	Effectiveness	Ineffective drug therapy	2 (1.6)
		Dose too low	24 (19.8)
	Safety	Dose too high	42 (34.7)
		Adverse drug reaction	3 (2.5)
	Non-compliance	Non-adherence	3 (2.5)
Total number of DRPs identified			121 (100)
Class of medications involved in DRPs		Antibiotics	52 (42.9)
		Analgesics-Antipyretics	36 (29.7)
		Antiepileptic	17 (14.0)
		Maintenance fluid	7 (5.7)
		Vitamin k	4 (3.3)
		Ferrous sulfate/folic acid	3 (2.4)
		Others	2 (1.6)
		Total	121 (100)

*DRPs* drug-related problems

### Determinant factors for the occurrence of DRPs

On bivariate analysis, on admission vomiting (*p* = 0.01) and taking antibiotics plus other medication (*p* = 0.03) were significantly associated with the occurrence of DRPs. A total of eight (8) variables were candidate for multivariate regression at *p* < 0.25. On multivariate analysis, taking antibiotics plus other medications (AOR = 5.2, 95%CI [1.2–22.0], *P* = 0.02) was a determinant factor for the occurrence of DRPs (Table 5).

**Table 5:** Factors determining the occurrence of DRPs among neonates with sepsis

Variables		Drug-related problem identified		COR[95%CI]	*p*-value	AOR[95%CI]	*p*-value
		Yes (***n*** = 98)	No (***n*** = 103)				
Age of the neonate in days (M ± SD)		14.6 ± 17.2	11.0 ± 15.3	0.9 [0.9–1.0]	0.12	1.0 [0.9–1.0]	0.80
Birth weight (kg) (M ± SD)		2.6 ± 0.7	2.8 ± 0.6	1.3 [0.8–2.0]	0.19	0.7 [0.4–1.2]	0.29
On admission vomiting	Yes	30 (30.6)	16 (15.5)	0.4 [0.2–0.8]	0.01	2.2 [0.98–4.9]	0.05
	No	68 (69.4)	87 (84.5)	1		1	
Hemoglobin (M ± SD)		13.7 ± 3.9	14.6 ± 3.5	1.0 [0.9–1.1]	0.09		
Oxygen saturation on admission (%)		79.1 ± 10.8	81.7 ± 11.7	1.0 [0.9–1.0]	0.11	0.9 [0.9–1.0]	0.25
Type of sepsis	EONS	55 (56.1%)	67 (65.0%)	1.4 [0.8–2.5]	0.19	0.8 [0.3–2.3]	0.79
	LONS	43 (43.9%)	36 (35.0%)	1		1	
Neonates taking antibiotics plus other medications	Yes	53 (54.1%)	40 (38.8%)	0.5 [0.3–0.9]	0.03	5.2 [1.2–22.0]	0.02
	No	45 (45.9%)	63 (61.2%)	1		1	
Number of medications taking during hospital stay (M ± SD)		2.6 ± 0.6	2.5 ± 0.7	0.7 [0.5–1.1]	0.21	0.4 [0.1–1.2]	0.11
Discharge status	Alive/discharged	71 (72.4)	85 (82.5)	1.7 [0.9–3.5]	0.08	0.5 [0.2–1.0]	0.06
	Death	27 (27.6)	18 (17.5)	1		1	

*M* mean, *SD* standard deviation, *EONS* Early Onset Neonatal Sepsis, *LONS* Late Onset Neonatal Sepsis

## Discussion

This pioneer observational study was undertaken among 201 neonates with sepsis to assess burden of DRPs at NICU. In about half of the study population (48.8%), at least one DRPs were identified. Dose too high was the frequently identified DRPs. Besides, antibiotics (42.8%) followed by analgesic-antipyretics (29.9%) and antiepileptic (13.7%) were the most commonly involved class of medications in DRPs.

A very few studies have evaluated DRPs in neonates admitted at NICU. The proportion of DRPs identified in this study (48.8%) was higher compared with other works on the pediatric/child population in Hong Kong 25.6% [15], and the United Kingdom (UK) and Saudi Arabia 45.2% [14]. This might be due to the difference in the management protocol across different countries as well as a difference in the study population (previous studies enrolled pediatrics population), the current study recruited high-risk populations (i.e. neonates and admitted at NICU) to have DRPs [11].

The current result is also lower in comparison with other studies in UK 51.2% [16] and Brazil 59.8% [17]-76.4% [31]. This variation could be due to differences in study population characteristics; the former study was conducted on children with kidney disease and the latter enrolled a relatively large study population (600 neonates), while the recent study in Brazil [31] included neonates with cardiac problems treated at NICU and took over 3 years. The disease comorbidities increase the risk of DRP occurrence and long study duration increase the probability of detecting DRPs [32, 33].

At the neonatal care unit, medication doses are commonly calculated based on newborns’ weight, which in turn varies with high frequency. Prescribing medications without proper checkup of the daily weight variation to calculate the dose is a possible source of dosing error that requires attention when managing newborns [34]. This will justify our findings that a dosing problem as a frequently identified DRPs.

Drug classes that were most often involved in DRPs in this study were antibiotics followed by analgesic-antipyretics. This finding is consistent with previous studies involving pediatrics in Brazil [17], United Kingdom and Saudi Arabia [14]. Neonates are susceptible to infection from pre-and post-natal exposure to micro-organisms. As a result of the immaturity of the immune system and prematurity, these infants are at significant risk of bacterial invasion and systemic infection [35]. They keep neonates treated with antibiotic drugs frequently and increase the likelihood of detecting the antibiotics drugs involved in DRPs. Moreover, the study population in the current study was neonates diagnosed with sepsis who, need antibiotic therapy.

Among neonates with DRPs in this study, antibiotic dosing contributed to 24.5% of a dose too high and 7.1% of a dose too low class of DRPs. Thus, clinicians have to seek more attention while calculating the dosage of medicines including antibiotics too for the neonate as a function of updated body weight.

According to the multivariate analysis, neonates taking antibiotics plus other medications during their hospital stay had the higher risks (more than five times) of DRPs compared with those taking only antibiotics. This is explicated by the pre-existing evidence showing the linear relationship between the number of medications taken and the occurrence of DRPs per patient [36, 37]. Furthermore, it probably implies that practitioners in our study setup give less attention to medical conditions and medication regimens, which were not the primary reason for the admission.

Despite this study is the first on assessing DRPs on neonates in Ethiopia as well as in Africa, it has some limitations. DRPs were analyzed concerning their potential health consequences, but its actual harm on the neonates was not assessed. We did not report the reasons for DRPs and make interventions for DRPs. The study included only one hospital, which may limit the generalization of the finding. Furthermore, this study lacks adequate comparison with previously conducted studies because of limited studies in similar areas.

## Conclusion

In conclusion, the burdens of DRPs occurrence were high in hospitalized neonates with sepsis. The most common DRPs identified were related to the dosing problem. Taking antibiotics plus other medications during their hospital stay was a determinant factor for the occurrence of DRPs. The innovative way to tackle the occurrence of DRPs, such as the incorporation of clinical pharmacy service provider into the neonatal care team, which will prevent, detect and/or minimize the occurrence of DRPs, is highly recommended. Besides, the authors recommended further studies with a large sample size to identify the problem on a large scale.

## Supplementary Information

**Additional file 1.**

## Data Availability

The datasets generated during and/or analyzed during the current study are available and submitted to the journal in the name of “Additional file 1”.

## Abbreviations

AOR: Adjusted Odds Ratio
COR: Crude Odds Ratio
CSF: Cerebrospinal Fluid
DRP: Drug-Related Problems
EONS: Early Onset Neonatal Sepsis
JUMC: Jimma University Medical Center
ICU: Intensive Care Unit
LONS: Late-Onset Neonatal Sepsis
NICU: Neonatal Intensive Care Unit
SD: Standard Deviation
SPSS: Statistical Package for Social Science
WHO: World Health Organizations
